# Impact of Fear of Falling on Future Falls and Changes in Physical Activity in Older Adults With Glaucoma

**DOI:** 10.1093/geroni/igaa057.2780

**Published:** 2020-12-16

**Authors:** Pradeep Ramulu, Jian-Yu E, Aleksandra Mihailovic, Pei-Lun Kuo, Sheila West, Laura Gitlin, Tianjing Li, Jennifer Schrack

**Affiliations:** 1 Johns Hopkins School of Medicine, Baltimore, Maryland, United States; 2 Johns hopkins Bloomberg School of Public Health, Baltimore, Maryland, United States; 3 Johns Hopkins University/Wilmer Eye Institute, Baltimore, Maryland, United States; 4 National Institute on Aging, Bethesda, Maryland, United States; 5 Drexel University, Philadelphia, Pennsylvania, United States; 6 University of Colorado, Arora, Colorado, United States; 7 Johns Hopkins Bloomberg School of Public Health, Baltimore, Maryland, United States

## Abstract

To understand how Fear of falling (FoF) alters mobility, FoF was evaluated annually in 243 older adults (median age=70) with varying degrees of visual field loss from glaucoma, and Rasch-analyzed FoF scores associated with the likelihood of falling in the following year (judged by prospective calendar data) and changes in physical activity (Judged by annual accelerometer trials). At lower FoF levels, each one-unit worsening in FoF was associated with a 2.73-fold higher odds of reporting a fall in the next year (95% CI:1.55,4.81) but not with average daily steps taken (p = 0.44). At higher FoF levels, inter-year changes in FoF were not significantly associated with a fall in the next year (p = 0.78); but were associated with 407 fewer daily steps taken per one-unit change in FoF (95% CI:-743,-71). FoF is an important driver of mobility; the specific aspects of mobility affected varies by the degree of FoF.

